# Draft genome sequence of mangrove-isolated fungus *Trichoderma reesei* MA2 reveals potential antifungal compounds and other secondary metabolites

**DOI:** 10.1128/mra.00720-25

**Published:** 2026-02-13

**Authors:** Alonso Segura-Valverde, Stefany Solano-González

**Affiliations:** 1 Laboratorio de Bioinformática Aplicada, Escuela de Ciencias Biológicas, Universidad Nacional 607742 , Heredia, Costa Rica; University of California Riverside, Riverside, California, USA

**Keywords:** secondary metabolites, BGC, Costa Rica, antiSMASH, ilicicolin, lignocellulolytic enzyme, BUSCO, genome assembly, genome structure

## Abstract

*Trichoderma reesei* MA2, isolated from Costarrican mangrove, exhibits biosynthetic potential to produce antifungal compounds and other secondary metabolites. The assembled genome was 33.4 Mb, with 9,510 protein-coding genes and 35 biosynthetic gene clusters, highlighting the potential of *T. reesei* MA2 as a source of unexplored bioactive compounds.

## ANNOUNCEMENT

Recently, biosynthetic gene clusters (BGCs) encoding hybrid polyketide synthase-nonribosomal peptide synthetases (PKS-NRPS) were identified in *Trichoderma reesei* (1, 2). These data revealed the production of ilicicolin H and K, both antifungal metabolites, sharing similar chemical properties and functioning as inhibitors of the mitochondrial respiratory chain (3). Here, we report the draft genome sequence, assembly, and annotation of *T. reesei* MA2 (4), isolated from mangrove sediment in Costa Rica.

Strains were isolated from Manuel Antonio National Park, Costa Rica. Ten grams of the collected sample was mixed with 90 mL of sterile filtered seawater (5 m) and shaken for 20 min at 150 rpm at room temperature. Serial dilutions were then made up to 10-2 in sterile filtered seawater. From each dilution, 100 µL per spread was inoculated in duplicate on potato dextrose agar (PDA) (BD) supplemented with 0.1 g/mL penicillin-streptomycin. The isolate was preserved as part of the collection of Laboratorio de Bioinformática Aplicada from Universidad Nacional. The species was confirmed through internal transcribed spacer (ITS) sequencing (GenBank accession number PP853646.1).

Isolate MA2 was grown in PDA for 7 days at 30°C in an orbital incubator (120 rpm), and DNA was extracted using the FastDNA Spin Kit for Soil (MP Biomedicals, Solon OH, USA) and a homogenizer (MM40, Retsch, Haan, Germany) according to the manufacturer’s protocol. Genome sequencing was performed using NovaSeq 6000 with the VAHTS Universal DNA Library Prep Kit for Illumina V4 platform in a paired-end 2 × 150 bp mode with a total of 6,195,146 paired-end sequence reads.

Adapters and low-quality sequences were trimmed using Trim Galore (v0.4.3) (5). *De novo* assembly was generated using SPAdes (v3.15.4) with a k-mer length of 89,95,97,101,107,117, and 127 with other default parameters (6). The assembly was improved with MeDusa scaffolder using *T. reesei* QM6a as reference (GCF_000167675.1) (7). Augustus (v3.3), with *Fusarium graminearum* as training set, was used to annotate proteins (8). tRNAs and rRNAs were predicted with tRNA-scan-SE (v2.0.5) and Barrnap (v0.9), respectively (9, 10). Genome completeness was assessed using Benchmarking Universal Single-Copy Orthologs (BUSCO) (v5.4.3) against the fungi_odb10, ascomycota_odb10, and hypocreales_odb10 databases (11). Analysis of BGCs encoding PKS-NRPS was performed using antiSMASH fungal version (v8.0) (12).

Sequencing yielded an average depth of 36.7×, and the final assembly resulted in 33.4 Mb, distributed in 259 scaffolds with an N50 of 1,068,146 bp and a GC content of 49.90% with 9,510 coding genes, 207 tRNAs, and 62 rRNA sequences. BUSCO results exhibited 99.0% (complete and single-copy BUSCOs [S]; 98.9%, complete and duplicated BUSCOs [D]: 0.1%), 98.6% (S: 98.4%, D: 0.2%), and 97.4% (S: 97.3%, D: 0.1%) of genome completeness against the Fungi (*n* = 758), Ascomycota (*n* = 1,706), and Hypocreales (*n* = 4,494) data sets, respectively. antiSMASH identified a total of 35 BGCs, comprising 11 terpene clusters, 10 NRPS clusters, eight type I PKS (T1PKS) clusters, and six hybrid clusters.

A T1PKS-NRPS cluster was found within scaffold 17 of the assembly (coordinates: nt 1208843–1274512), showing high similarity to the MIBiG database entry BGC0002035, which corresponds to the ilicicolin H BGC from *Neonectria* sp. DH2 (13). Furthermore, two additional hybrid BGCs, the NRPS-T1PKS cluster on scaffold 3 (coordinates: nt 279802–389733) and the T1PKS-NRPS-terpene cluster on scaffold 6 (coordinates: nt 1–111042), showed no similarity to any previously reported BGCs in the MIBiG database.

Our results underscore the potential capacity of *T. reesei* MA2 to produce novel antifungal agents and enhance its value as a source of secondary metabolites of biotechnological interest, complementing its well-established industrial role as producer of lignocellulolytic enzymes.

## Data Availability

The whole-genome shotgun project has been deposited in the GenBank database under the BioSample accession number PRJNA1278790 and at DDBJ/ENA/GenBank under the accession JBQXYM000000000. The version described in this paper is version JBQXYM010000000. The SRA data are available under the accession SAMN49246600.
